# The Triad of Sleep, Immunity, and Cancer: A Mediating Perspective

**DOI:** 10.3390/cells13151246

**Published:** 2024-07-24

**Authors:** Giuseppe Lanza, Maria P. Mogavero, Michele Salemi, Raffaele Ferri

**Affiliations:** 1 Oasi Research Institute—IRCCS, 94018 Troina, Italy; glanza@oasi.en.it (G.L.); msalemi@oasi.en.it (M.S.); 2 Department of Surgery and Medical–Surgical Specialties, University of Catania, 95100 Catania, Italy; 3 Vita-Salute San Raffaele University, 20132 Milan, Italy; paola_mogavero@libero.it; 4 Division of Neuroscience, Sleep Disorders Center, San Raffaele Scientific Institute, 20127 Milan, Italy

**Keywords:** sleep, immunity, cancer, immune regulation, tumor microenvironment, sleep interventions, sleep disorders, immune surveillance

## Abstract

The triadic interplay between sleep, immunity, and cancer represents a growing area of biomedical research with significant clinical implications. This review synthesizes the current knowledge on how sleep influences immune function, the immune system’s role in cancer dynamics, and the direct connections between sleep patterns and cancer risk. After a comprehensive overview of the interrelationships among these three domains, the mechanisms of sleep in immune function are described, detailing how sleep regulates the immune system, the effects of sleep duration and quality on immune responses, and the underlying molecular and cellular mechanisms. Also, the complex relationship between immunity and cancer is explored, highlighting the immune system’s role in cancer prevention and progression, immune surveillance, tumor microenvironment, and the implications of immunodeficiency and immune modulation on cancer risk. The direct connections between sleep and cancer are then described, presenting epidemiological evidence linking sleep patterns to cancer risk, biological mechanisms that influence cancer development, and the role of sleep disorders in cancer prognosis. The mediating role of sleep between immunity and cancer is highlighted, proposing hypothesized pathways, summarizing evidence from experimental and clinical studies, and evaluating the impact of sleep interventions on immune function and cancer outcomes. This review concludes by discussing the clinical implications and future directions, emphasizing the potential for sleep-based interventions in cancer prevention and treatment, the integration of sleep management in oncology and immunotherapy, and outlining a future research agenda. This agenda includes understanding the mechanisms of the sleep–immunity–cancer interplay, conducting epidemiological studies on sleep and cancer risk, assessing the impact of sleep management in cancer treatment protocols, exploring sleep and tumor microenvironment interactions, and considering policy and public health implications. Through a detailed examination of these interconnected pathways, this review underscores the critical importance of sleep in modulating immune function and cancer outcomes, advocating for interdisciplinary research and clinical strategies to harness this knowledge for improved health outcomes.

## 1. Introduction

In recent decades, the complex relationship between sleep, immunity, and cancer has garnered significant attention. Sleep disorders have been increasingly recognized not just as a consequence of cancer and its treatments, but also as a potential factor influencing cancer development and progression. Many studies have highlighted the bidirectional connection between sleep disturbances and cancer, noting that patients with various types of tumors often suffer from sleep disorders even before commencing treatments like chemotherapy or radiotherapy. Insomnia, hypersomnia, and circadian rhythm disorders are notably prevalent among cancer patients, impacting their quality of life and potentially contributing to cancer progression [1,2,3,4].

The interaction between sleep and cancer is further complicated by the role of the immune system. Adequate sleep is essential for maintaining robust immune function, which in turn plays a critical role in cancer prevention through mechanisms such as immune surveillance [5]. Conversely, poor sleep can lead to immune dysregulation, fostering an environment conducive to tumor growth and metastasis. Molecular mechanisms involving cytokines, neurotransmitters, and the hypothalamic–pituitary–adrenal (HPA) axis are central to this complex interplay, highlighting the need for a comprehensive understanding of how sleep influences immunity and cancer dynamics [6,7].

This narrative review aims to synthesize the latest research on the mediating role of sleep between immunity and cancer, highlighting key findings and exploring therapeutic implications. By examining the pathways through which sleep affects immune function and cancer outcomes, this review seeks to provide insights into potential interventions that could enhance cancer prevention and treatment through improved sleep health in young and adult individuals. 

Furthermore, modern lifestyle factors, including increased stress and irregular sleep patterns, have exacerbated the prevalence of sleep disturbances, posing additional challenges to immune function and cancer dynamics [8,9]. The role of circadian rhythms in regulating the immune response and maintaining cellular homeostasis underscores the importance of healthy sleep patterns [10]. Disrupted sleep can lead to immune dysregulation, increasing susceptibility to infections and potentially facilitating cancer development and progression. By examining both the natural regulation of sleep and the consequences of sleep disturbances, this review aims to provide a holistic understanding of how improving sleep health can serve as a critical component in cancer prevention and treatment strategies. Through this comprehensive approach, we hope to identify actionable insights and therapeutic interventions that enhance patient outcomes and quality of life.

## 2. Mechanisms of Sleep in Immune Function

### 2.1. Role of Sleep in Immune System Regulation

Sleep plays a crucial role in the regulation of the immune system, acting as a fundamental process for maintaining immune homeostasis and enhancing the body’s defense mechanisms [10,11]. During sleep, particularly during slow-wave sleep, the production and release of cytokines increase [12]. These cytokines, such as interleukin-1 (IL-1) and tumor necrosis factor (TNF), are essential for the initiation and coordination of immune responses. Sleep also facilitates the proliferation of T cells, a type of white blood cell that is critical for identifying and eliminating pathogens [13]. Consequently, adequate sleep is essential for the optimal functioning of both the innate and adaptive immune responses, ensuring the body is prepared to fight off infections and diseases.

Furthermore, sleep influences the balance between pro-inflammatory and anti-inflammatory responses, which is vital for preventing chronic inflammation and autoimmune conditions. Research indicates that during sleep, sympathetic nervous system activity decreases and parasympathetic activity increases, leading to reduced levels of stress hormones such as cortisol [14]. Lower cortisol levels during sleep allow the immune system to function more efficiently and reduce the risk of inflammatory diseases. Additionally, sleep enhances immunological memory by strengthening the interaction between dendritic cells and T cells, improving the body’s ability to recognize and respond more effectively to previously encountered pathogens [15]. Thus, regular and restorative sleep is essential for maintaining a well-regulated and responsive immune system.

### 2.2. Impact of Sleep Duration and Quality on Immune Responses

Sleep duration and quality have profound impacts on immune responses, as evidenced by multiple studies. Short sleep duration, typically defined as less than seven hours per night, has been consistently linked to impaired immune function [16]. For instance, individuals who consistently get insufficient sleep exhibit a higher susceptibility to infections, such as the common cold. This is because inadequate sleep reduces the production of cytokines, proteins that play a crucial role in immune response by targeting infection and inflammation. Additionally, sleep deprivation is associated with lower levels of infection-fighting antibodies and cells, leading to a weakened immune defense.

Moreover, sleep quality, not just duration, significantly influences immune function. Poor sleep quality, characterized by frequent awakenings and non-restorative sleep, is associated with increased inflammation and dysregulation of the immune system. Studies have shown that individuals with poor sleep quality have higher levels of pro-inflammatory markers such as C-reactive protein (CRP) and IL-6 [16]. These markers are indicative of chronic low-grade inflammation, which can contribute to a variety of health issues including cardiovascular diseases and autoimmune disorders. Therefore, both adequate sleep duration and high sleep quality are essential for maintaining a robust and effective immune system.

### 2.3. Molecular and Cellular Mechanisms Linking Sleep and Immunity

The molecular and cellular mechanisms linking sleep and immunity involve intricate interactions between various immune cells, signaling pathways, and hormonal changes. At the cellular level, sleep enhances the activity of T cells, which are essential for adaptive immunity [15,17]. During sleep, there is an increased expression of integrins on T cells, which are proteins that facilitate the cells’ ability to attach to and destroy infected cells. Additionally, sleep promotes the redistribution of immune cells, such as monocytes and natural killer (NK) cells, to lymphoid tissues, enhancing the body’s ability to detect and respond to pathogens [18].

Molecularly, sleep regulates the production and release of cytokines, which are crucial signaling molecules in the immune system. For example, during deep sleep, levels of pro-inflammatory cytokines such as IL-1 and TNF-α increase, promoting immune activation and pathogen defense [15]. Conversely, sleep deprivation can lead to a decrease in these cytokines, impairing immune function [17]. Furthermore, sleep affects the HPA axis, reducing the secretion of cortisol, a stress hormone that can suppress immune activity when elevated [14]. This reduction in cortisol during sleep allows for a more robust immune response, facilitating the repair and regeneration of tissues and enhancing overall immune function. These molecular and cellular interactions underscore the essential role of sleep in maintaining a resilient and responsive immune system.

## 3. Immunity and Cancer: A Complex Relationship

### 3.1. Immune System’s Role in Cancer Prevention and Progression

The immune system plays a crucial role in cancer prevention and progression through its ability to detect and eliminate malignant cells. One of the primary mechanisms by which the immune system prevents cancer is through immune surveillance [19]. Immune surveillance involves the continuous monitoring of cells by the immune system to detect and destroy abnormal cells before they can develop into cancer. Key players in this process include cytotoxic T lymphocytes (CTLs) and NK cells [20]. CTLs recognize and kill cells presenting abnormal antigens via major histocompatibility complex (MHC) class I molecules, while NK cells can directly kill cells that lack MHC class I molecules or are otherwise stressed. This dynamic interplay helps prevent the establishment and growth of tumors in their early stages.

However, once a tumor is established, the immune system continues to influence cancer progression, both positively and negatively. On the one hand, the immune system can suppress tumor growth through the actions of CTLs, NK cells, and macrophages, which target and destroy cancer cells. On the other hand, tumors can exploit various immune evasion strategies to avoid detection and destruction [20]. For instance, tumors may downregulate MHC class I molecules, produce immunosuppressive cytokines like transforming growth factor-beta (TGF-β) and IL-10, or recruit regulatory T cells (Tregs) to create an immunosuppressive microenvironment [21,22]. This immunosuppressive environment can inhibit the activity of CTLs and NK cells, allowing the tumor to grow and metastasize.

The dual role of the immune system in cancer prevention and progression has significant implications for cancer therapy. Immunotherapy, which aims to enhance the body’s immune response against cancer, has emerged as a promising treatment approach [23]. Checkpoint inhibitors, such as pembrolizumab and nivolumab, work by blocking inhibitory signals on T cells, thereby enhancing their ability to attack cancer cells [24]. Other strategies include adoptive cell transfer, where patients’ own immune cells are modified and reinfused to better target cancer, and cancer vaccines, which stimulate the immune system to recognize and attack tumor-specific antigens [25]. These therapies underscore the importance of the immune system in both preventing and controlling cancer, highlighting the potential for harnessing immune mechanisms to improve cancer outcomes.

### 3.2. Immune Surveillance and Tumor Microenvironment

The tumor microenvironment (TME) profoundly influences cancer progression by creating conditions that can suppress immune responses and promote tumor growth. The TME comprises not only cancer cells but also stromal cells, immune cells, and extracellular matrix components, forming a complex ecosystem that interacts with and modulates the behavior of tumor cells [26]. Tumors can alter the TME to their advantage by recruiting immunosuppressive cells such as Tregs and myeloid-derived suppressor cells, which inhibit the activity of effector immune cells [27]. Additionally, the secretion of immunosuppressive cytokines like TGF-β and IL-10 further dampens immune responses [28]. These alterations enable tumor cells to evade immune surveillance, support angiogenesis, and facilitate metastasis. Understanding these interactions is critical for developing therapeutic strategies aimed at modulating the TME to enhance anti-tumor immunity and improve cancer treatment outcomes.

### 3.3. Effects of Immunodeficiency and Immune Modulation on Cancer Risk

Immunodeficiency significantly increases the risk of cancer due to an impaired ability of the immune system to perform effective immune surveillance and eliminate emerging malignant cells [29,30]. Individuals with primary immunodeficiency disorders, such as severe combined immunodeficiency or common variable immunodeficiency, have a higher incidence of cancers, particularly lymphomas and leukemias [31,32]. Similarly, acquired immunodeficiencies, such as those caused by human immunodeficiency virus infection or immunosuppressive treatments (e.g., chemotherapy or organ transplant medications), also elevate cancer risk [33]. In these conditions, the reduction in functional immune cells, such as T cells and NK cells, diminishes the body’s capacity to detect and destroy cancerous cells, allowing tumors to grow unchecked.

Immune modulation, whether through pharmacological agents or lifestyle interventions, can alter cancer risk by either enhancing or suppressing immune function. Immunosuppressive drugs, commonly used to prevent transplant rejection or treat autoimmune diseases, can inadvertently increase cancer risk by dampening the immune system’s ability to combat tumor cells [34,35]. Conversely, immune-enhancing strategies, such as the use of immune checkpoint inhibitors in cancer therapy, can boost the immune system’s capacity to target and eliminate cancer cells, reducing the likelihood of tumor progression [36]. Lifestyle factors like diet, exercise, and stress management also play a role in modulating immune function. For instance, chronic stress and poor nutrition can weaken immune responses, potentially increasing cancer susceptibility, while regular exercise and a balanced diet can bolster immune defenses, contributing to cancer prevention [37,38].

## 4. Sleep and Cancer: Direct Connections

### 4.1. Epidemiological Studies Linking Sleep Patterns and Cancer Risk

Epidemiological studies have established significant links between sleep patterns and cancer risk, highlighting the importance of adequate and quality sleep for cancer prevention. Research indicates that insufficient sleep, typically defined as less than six to seven hours per night, is associated with an increased risk of several types of cancer, including breast, prostate, and colorectal cancers [39,40]. For instance, a large cohort study found that women who slept less than six hours per night had a significantly higher risk of breast cancer compared to those who slept seven to eight hours [41]. Similar findings have been reported in men, where short sleep duration has been linked to a higher risk of prostate cancer [42,43]. These studies suggest that chronic sleep deprivation may disrupt circadian rhythms and hormonal balances, contributing to cancer development.

Moreover, irregular sleep patterns and shift work, which often lead to circadian rhythm disruption, have also been associated with increased cancer risk. The International Agency for Research on Cancer (IARC) has classified shift work that involves circadian disruption as a probable carcinogen [44]. Epidemiological evidence supports this classification, showing higher incidences of breast, prostate, and colorectal cancers among night shift workers [45,46]. For example, a study found that women who worked night shifts for several years had a higher risk of breast cancer compared to those who did not engage in shift work. The underlying mechanisms are believed to involve the suppression of melatonin, a hormone with anti-cancer properties, and the dysregulation of genes involved in cell cycle control and DNA repair [47]. These findings underscore the importance of maintaining regular sleep patterns and minimizing circadian disruption to reduce cancer risk.

### 4.2. Biological Mechanisms through Which Sleep Influences Cancer Development

Sleep influences cancer development through several complex biological mechanisms that affect cellular processes and systemic hormonal regulation [48]. One of the primary ways sleep impacts cancer risk is by regulating the circadian rhythm, which controls various physiological functions including cell cycle progression and DNA repair. Disruptions in circadian rhythms, often caused by insufficient or irregular sleep, can lead to altered expression of clock genes. These genes are crucial for maintaining cellular homeostasis, and their dysregulation can promote uncontrolled cell proliferation and tumor growth [49]. For example, the suppression of the production of melatonin, a hormone that is typically elevated during night sleep, can impair its oncostatic properties. Melatonin acts as an antioxidant and regulates estrogen receptor expression; thus, its reduced levels can enhance the risk of hormone-related cancers like breast and prostate cancer [50]. On the other hand, an upregulation of estrogen receptors has been found in periodic leg movements during sleep (PLMS) [51]. Sleep-related movement disorders are often characterized by a marked fragmentation of sleep, and PLMS in particular are sometimes associated with some tumors, including breast cancer [2].

Another key mechanism is the modulation of immune function by sleep [52]. Adequate sleep is essential for the optimal functioning of the immune system, which includes the activity of CTLs and NK cells that target and destroy cancer cells. Sleep deprivation or poor sleep quality can lead to a decline in the production of these immune cells and cytokines that facilitate their actions [53]. Chronic sleep disturbances can result in a persistent inflammatory state, characterized by elevated levels of pro-inflammatory cytokines such as IL-6 and TNF-α [54]. This inflammatory milieu can contribute to an environment conducive to cancer development by promoting DNA damage, facilitating tumor growth, and enhancing metastatic potential. Thus, maintaining regular and restorative sleep is crucial for sustaining effective immune surveillance and minimizing cancer risk.

### 4.3. Role of Sleep Disorders in Cancer Prognosis

Sleep disorders can significantly impact cancer prognosis by influencing treatment outcomes, quality of life, and overall survival rates. Patients with cancer often experience sleep disturbances, including insomnia, sleep apnea, and circadian rhythm disruptions, which can exacerbate symptoms such as fatigue, pain, and emotional distress [2,55]. Poor sleep quality and insufficient sleep duration have been associated with reduced tolerance to cancer treatments such as chemotherapy and radiation therapy [56]. These treatments can further disrupt sleep patterns, creating a cycle that negatively impacts both physical and psychological well-being [57,58]. Moreover, sleep disorders can compromise immune function, impairing the body’s ability to effectively combat cancer cells and recover from treatment-related side effects [59].

Furthermore, the relationship between sleep disorders and cancer prognosis extends beyond treatment tolerance to affect overall survival rates. Studies have shown that cancer patients with untreated sleep disorders may have poorer outcomes compared to those with adequate sleep. For instance, sleep disturbances have been linked to increased inflammation, which can promote tumor progression and metastasis [60,61]. Additionally, the hormonal disruptions caused by sleep disorders, such as alterations in melatonin production, may contribute to tumor growth and resistance to treatment [62,63]. These studies underscore the importance of addressing sleep disturbances as a potential modifiable risk factor in cancer prevention and management. As such, addressing sleep disorders through appropriate interventions, such as cognitive–behavioral therapy for insomnia (CBT-I) [64], continuous positive airway pressure (CPAP) therapy for sleep apnea, or pharmacological treatments, is essential not only for improving quality of life but also for optimizing cancer treatment efficacy and potentially enhancing survival outcomes [2].

## 5. The Mediating Role of Sleep between Immunity and Cancer

### 5.1. Hypothesized Pathways of Mediation

Sleep disorders may mediate the relationship between immunity and cancer through several hypothesized pathways, each influencing immune function and potentially impacting cancer development. Inflammatory cytokines such as IL-1β, IL-6, and TNF-α, which are elevated in both sleep disorders and cancer, play a critical role in immune dysregulation [65,66]. These cytokines contribute to chronic inflammation, which can suppress immune responses and create a microenvironment favorable for tumor growth and metastasis. For example, prolonged exposure to elevated IL-6 levels in sleep disorders may disrupt the balance between pro-inflammatory and anti-inflammatory responses, thereby impairing the immune system’s ability to effectively recognize and eliminate cancer cells [67,68].

Additionally, disturbances in sleep architecture and circadian rhythms can lead to alterations in hormone production, including melatonin and cortisol, which further impact immune function and cancer susceptibility. Melatonin, primarily synthesized during nighttime sleep, exhibits potent antioxidant and immunomodulatory properties. Reduced melatonin levels due to sleep disorders may diminish its anti-cancer effects, such as inhibiting tumor cell proliferation and promoting apoptosis [69]. Moreover, disruptions in the circadian rhythm, often observed in shift workers or individuals with irregular sleep patterns, can lead to dysregulated cortisol secretion. Elevated cortisol levels, particularly during nighttime sleep disruption, have been associated with immune suppression and increased inflammation, potentially fostering conditions conducive to cancer progression [70].

Interventions aimed at improving sleep quality have shown promise in mitigating these pathways and potentially reducing cancer risk. For instance, therapies such as CBT-I aim to restore healthy sleep patterns and have been associated with improvements in immune function [71]. Studies suggest that restoring adequate sleep duration and quality may enhance the activity of immune cells, including NK cells and CTLs, thereby bolstering immune surveillance against cancer [72]. Furthermore, treatments like CPAP for obstructive sleep apnea not only alleviate sleep fragmentation but also reduce levels of inflammatory markers and improve vascular health, which are crucial factors in tumor angiogenesis and growth [73]. These insights highlight the interconnectedness of sleep, immune function, and cancer biology, underscoring the importance of addressing sleep disorders in cancer prevention and treatment strategies.

### 5.2. Evidence from Experimental and Clinical Studies

Experimental and clinical studies provide compelling evidence for the mediating role of sleep between immunity and cancer, elucidating various mechanisms through which sleep influences immune function and cancer outcomes. Research has demonstrated that sleep disturbances, such as chronic sleep deprivation or fragmented sleep, can dysregulate immune responses by altering the production of cytokines and immune cells [74]. For instance, experimental studies have shown that sleep deprivation in animal models leads to increased levels of pro-inflammatory cytokines like IL-6 and TNF-α, which can suppress immune surveillance and promote tumor growth [75]. These cytokines not only modulate local inflammation but also impact systemic immune function, potentially compromising the body’s ability to recognize and eliminate cancer cells.

Clinical studies have further corroborated these findings by linking poor sleep quality and duration with impaired immune responses and increased cancer risk. For example, epidemiological investigations have found associations between short sleep duration and elevated markers of systemic inflammation, such as CRP, which are implicated in cancer progression [67]. Furthermore, intervention studies exploring the effects of improving sleep quality on immune function and cancer-related outcomes have yielded promising results. For instance, clinical trials evaluating the efficacy of sleep interventions, such as CBT-I or CPAP therapy for sleep apnea, have reported improvements in immune parameters and reduced inflammation markers [76,77]. These interventions not only promote better sleep hygiene but also enhance immune surveillance mechanisms that play a crucial role in detecting and eliminating cancer cells. By targeting sleep as a mediator between immunity and cancer, these studies highlight the potential for integrated approaches that incorporate sleep management strategies into comprehensive cancer care plans to improve patient outcomes.

## 6. Clinical Implications and Future Directions

### 6.1. Potential for Sleep-Based Interventions in Cancer Prevention and Treatment

Sleep-based interventions hold significant potential in both cancer prevention and treatment strategies, offering novel avenues to improve patient outcomes and quality of life. Prevention efforts focus on addressing sleep disturbances as modifiable risk factors for cancer development. Epidemiological studies consistently link inadequate sleep duration and poor sleep quality with increased cancer risk, highlighting the importance of promoting healthy sleep habits from an early age. By implementing educational programs and interventions that emphasize the importance of adequate sleep, healthcare providers can potentially reduce the incidence of sleep-related risk factors associated with cancer [78].

In cancer treatment, sleep-based interventions play a crucial role in enhancing therapeutic efficacy and mitigating treatment-related side effects. For instance, optimizing sleep hygiene through behavioral interventions like CBT-I can improve sleep quality and reduce symptoms of sleep disorders commonly experienced by cancer patients [79,80]. Improved sleep hygiene not only enhances immune function but also supports overall physical and psychological well-being, potentially improving treatment tolerance and adherence [81]. Additionally, interventions such as CPAP therapy for obstructive sleep apnea have shown promise in reducing inflammation and oxidative stress, which are critical factors in cancer progression [82]. Integrating these sleep-based interventions into comprehensive cancer care plans can contribute to personalized treatment approaches that optimize outcomes and enhance the quality of life for cancer patients.

In addition to behavioral interventions, pharmacological treatments also offer potential avenues for addressing sleep disturbances in cancer patients. Pharmacotherapy may be considered when behavioral interventions alone are insufficient or when rapid improvement in sleep is needed. Medications such as sedative–hypnotic drugs, including benzodiazepines and non-benzodiazepine hypnotics, are commonly prescribed to improve sleep onset and maintenance. These medications can be particularly useful for managing acute insomnia related to cancer treatment regimens or hospitalization [83].

Orexin agonists and antagonists are a class of medications that target the orexin system in the brain, which plays a crucial role in regulating wakefulness and sleep. Orexins, also known as hypocretins, are neuropeptides that promote wakefulness and arousal by influencing various neurotransmitter systems [84]. Agonists of the orexin receptors, such as suvorexant, work by blocking the binding of orexins to their receptors, thereby promoting sleep initiation and maintenance. These medications are particularly effective in treating insomnia characterized by difficulty falling or staying asleep [85]. On the other hand, orexin antagonists, which enhance the inhibitory effects of orexin neurons, can be used to promote sleep by reducing wakefulness-promoting signals [86]. Both orexin agonists and antagonists represent innovative approaches to managing sleep disorders and may hold promise in addressing insomnia and other sleep disturbances in cancer patients, potentially improving their overall quality of life and treatment outcomes [87].

Furthermore, medications targeting specific sleep disorders, such as melatonin agonists for circadian rhythm disorders or stimulants for excessive daytime sleepiness, can be tailored to the individual needs of cancer patients. For instance, melatonin supplementation has been studied for its potential role in improving sleep quality and regulating circadian rhythms disrupted by cancer treatments or shift work [88]. By addressing sleep disturbances pharmacologically, healthcare providers can complement behavioral interventions and enhance the effectiveness of multidisciplinary approaches to cancer care. However, careful consideration of potential side effects, drug interactions, and individual patient characteristics is essential to ensure safe and effective use of pharmacological treatments for sleep disorders in cancer patients.

### 6.2. Integrating Sleep Management in Oncology and Immunotherapy

Integrating sleep management into oncology and immunotherapy practices is increasingly recognized as a critical component of comprehensive cancer care. Sleep disturbances are prevalent among cancer patients and can significantly impact treatment outcomes, immune function, and overall quality of life. Therefore, incorporating routine screening for sleep disorders and implementing evidence-based sleep interventions are essential steps in optimizing cancer treatment strategies. For instance, oncology clinics can adopt multidisciplinary approaches that include sleep specialists who collaborate with oncologists to assess and manage sleep-related issues throughout the cancer care continuum [89]. This collaboration ensures that patients receive tailored sleep interventions that complement their cancer treatment plans, potentially improving treatment adherence and outcomes.

In the context of immunotherapy, sleep management becomes even more pertinent due to the interplay between sleep, immune function, and cancer progression. Immunotherapy aims to enhance the body’s immune response against cancer cells, and adequate sleep plays a crucial role in maintaining optimal immune function. Studies have shown that poor sleep quality and duration can compromise immune responses, potentially reducing the effectiveness of immunotherapy treatments [90]. Therefore, integrating sleep management strategies such as behavioral interventions (e.g., CBT-I) or pharmacological treatments into immunotherapy protocols may help optimize immune function and enhance treatment efficacy. By addressing sleep disturbances as part of a comprehensive cancer care plan, healthcare providers can potentially improve patient outcomes, minimize treatment-related complications, and enhance the overall well-being of cancer patients undergoing immunotherapy.

### 6.3. The Impact of Pharmacologically Induced Sleep on Immunity and Cancer Risk

In recent years, the prevalence of sleep disturbances has led to a significant increase in the use of sleeping pills, such as benzodiazepines and non-benzodiazepine hypnotics. These medications, while effective in inducing sleep, alter the natural architecture of sleep by reducing the time spent in the deep, restorative sleep stages [91,92]. This disruption can have notable effects on immune function, which is closely tied to the quality of sleep. The immune system relies on sleep to maintain its homeostasis and efficiency; hence, altered sleep patterns due to sleeping pills can impair immune responses. Furthermore, emerging research suggests a potential link between the long-term use of these medications and an increased risk of cancer [93]. The immunomodulatory effects of sleeping pills, including their impact on cytokine production and natural killer cell activity, may contribute to this increased risk [94]. Understanding the complex relationship between pharmacologically induced sleep, immunity, and cancer is crucial, especially in the context of addressing sleep health in modern society. This discussion underscores the importance of considering the broader implications of sleeping pill use beyond their immediate benefits for sleep induction.

### 6.4. Interconnections between Sleep Quality, Diet, Lifestyle and Cancer

Sleep quality is influenced by a myriad of factors including stress levels, sleep environment (such as noise, light, and temperature), and lifestyle habits like exercise and screen time before bed [95]. Diets high in processed foods, sugars, and unhealthy fats can contribute to poor sleep quality, reduced immunity, and an increased risk of cancer [96]. Conversely, a diet rich in fruits, vegetables, lean proteins, and whole grains can enhance sleep quality by providing essential nutrients that support the body’s natural sleep–wake cycle [97]. Environmental and lifestyle changes such as exposure to blue light from electronic devices, irregular sleep schedules, and high levels of stress can disrupt sleep and elevate cancer risk [98]. On a molecular level, quality sleep supports cellular repair and regulation of hormonal balance, which can be influenced by diet and lifestyle.

**Figure 1:**
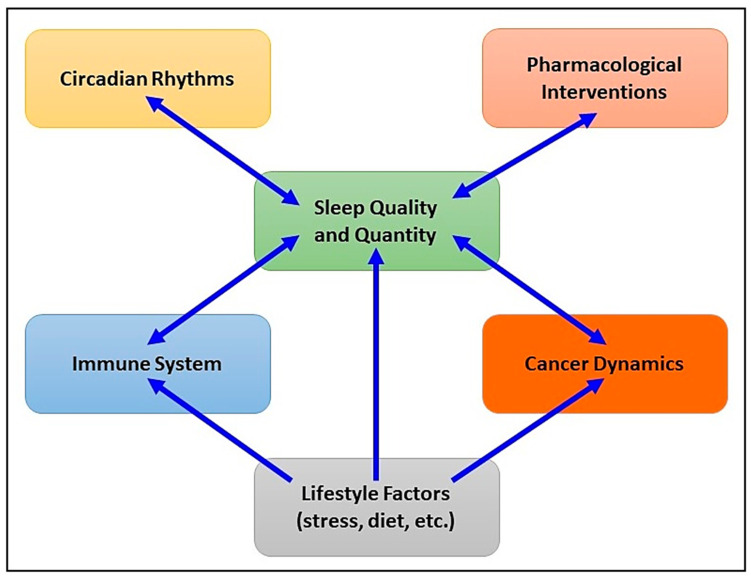
Comprehensive visual representation of how sleep mediates the relationship between immune function and cancer development, influenced by lifestyle factors and pharmacological interventions.

Figure 1 offers a comprehensive visual representation of how sleep mediates the relationship between immune function (including specific components like cytokine production, immune cell function, and inflammation) and cancer (tumor growth, metastasis, and response to treatment), influenced by lifestyle factors (stress, diet, physical activity, and environmental factors) and pharmacological interventions.

### 6.5. Research Agenda

#### 6.5.1. Understanding Mechanisms of Interplay between Sleep, Immunity, and Cancer 

To elucidate the molecular and cellular pathways through which sleep disturbances influence immune function and cancer development, we make the following recommendations for future research:

- Study the impact of sleep disturbances on the production and regulation of pro-inflammatory cytokines (e.g., IL-1β, IL-6, TNF-α) and their roles in immune suppression and tumor promotion.
- Examine how disruptions in sleep affect the secretion of hormones like melatonin and cortisol and their subsequent effects on immune function and cancer cell proliferation.
- Assess how sleep quality impacts the activity and efficacy of various immune cells, including NK cells, CTLs, and regulatory T cells, in cancer surveillance and elimination.

#### 6.5.2. Epidemiological Studies on Sleep and Cancer Risk

To establish population-level associations between sleep patterns, sleep disorders, and cancer incidence, we make the following recommendations for future research:

- Conduct long-term studies tracking sleep habits and cancer development, controlling for confounding factors such as lifestyle, genetic pre-dispositions, and environmental exposures.
- Aggregate data from existing studies to identify consistent patterns and risk factors linking sleep disturbances with specific types of cancer.
- Evaluate the differential impact of sleep disorders on various demographic groups (e.g., age, gender, ethnicity) to identify vulnerable populations.

#### 6.5.3. Interventional Studies on Sleep Improvement and Cancer Outcomes

To determine the efficacy of sleep interventions in enhancing immune function and improving cancer prognosis, we make the following recommendations for future research:

- Design and implement randomized controlled trials (RCTs) testing the effects of sleep interventions, such as CBT-I, CPAP therapy, and pharmacological treatments, on immune markers and cancer outcomes.
- Explore the combined effects of behavioral and pharmacological treatments on sleep quality and their synergistic impact on cancer treatment efficacy.
- Incorporate assessments of patient-reported sleep quality, fatigue, and overall well-being in interventional studies to capture holistic benefits.

#### 6.5.4. Impact of Sleep Management in Cancer Treatment Protocols

To integrate sleep management into standard oncology care and assess its impact on treatment adherence and patient quality of life, we make the following recommendations for future research:

- Implementation studies: develop and test protocols for routine sleep disorder screening and management in oncology settings.
- Quality-of-life measures: evaluate how sleep management influences patient-reported outcomes related to fatigue, pain, mental health, and overall quality of life during cancer treatment.
- Treatment adherence and efficacy: assess whether improving sleep quality leads to better adherence to cancer treatments (e.g., chemotherapy, immunotherapy) and enhances their efficacy.

#### 6.5.5. Exploration of Sleep and Tumor Microenvironment Interactions

To understand how sleep disturbances affect the tumor microenvironment (TME) and its interactions with immune cells, we make the following recommendations for future research:

- Investigate how sleep-induced changes in the TME influence cancer cell proliferation, angiogenesis, and metastatic potential.
- Study the impact of sleep quality on the infiltration and activity of immune cells within the TME.
- Use advanced techniques such as single-cell RNA sequencing to profile the TME under different sleep conditions.

#### 6.5.6. Policy and Public Health Implications

To translate research findings into public health policies and clinical guidelines promoting sleep health as a component of cancer prevention and care, we make the following recommendations for future research:

- Develop educational campaigns highlighting the importance of sleep for cancer prevention and general health.
- Create evidence-based guidelines for healthcare providers on integrating sleep management into cancer care protocols.
- Analyze the cost-effectiveness of sleep interventions in cancer care to support policy decisions and funding allocations.

## 7. Conclusions

The very complex relationship between sleep, immune function, and cancer has been highlighted through various studies, underscoring the significant impact that sleep disturbances can have on cancer development and progression. Key findings indicate that inadequate sleep and sleep disorders, such as insomnia and obstructive sleep apnea, are associated with elevated levels of pro-inflammatory cytokines like IL-6 and TNF-α, which can impair immune surveillance and promote tumor growth. Interventions aimed at improving sleep quality, including CBT-I, CPAP therapy for sleep apnea, and pharmacological treatments have shown promise in reducing inflammation and enhancing immune responses, thereby potentially improving cancer outcomes.

The integration of sleep management into oncology and immunotherapy protocols has significant implications for clinical practice and public health. Routine screening for sleep disorders should become a standard part of cancer care to identify and address sleep disturbances early in the treatment process. Implementing evidence-based sleep interventions can improve treatment adherence, reduce side effects, and enhance overall patient well-being. Public health initiatives should emphasize the importance of adequate sleep for cancer prevention, advocating for educational programs and lifestyle modifications that promote healthy sleep habits. By recognizing sleep as a crucial component of cancer care, healthcare providers can adopt a more holistic approach that optimizes patient outcomes.

The importance of considering sleep in the context of immunity and cancer cannot be overstated. Sleep is a fundamental biological process that influences numerous physiological systems, including the immune system’s ability to detect and eliminate cancer cells. Future research should continue to explore the underlying mechanisms of the sleep–immune–cancer triad and evaluate the long-term benefits of sleep interventions in cancer prevention and treatment. By advancing our understanding and addressing sleep disturbances through targeted interventions, we can significantly enhance the effectiveness of cancer therapies and improve the quality of life for cancer patients. Prioritizing sleep health is not only vital for individual well-being but also a critical aspect of comprehensive cancer care and public health strategies.
